# Cabozantinib in combination with immune checkpoint inhibitors for renal cell carcinoma: a systematic review and meta-analysis

**DOI:** 10.3389/fphar.2024.1322473

**Published:** 2024-04-17

**Authors:** Jingyang Su, Jialin Zhang, Yuqian Wu, Cui Ni, Yueyue Ding, Zelin Cai, Ming Xu, Mingyang Lai, Jue Wang, Shengyou Lin, Jinhua Lu

**Affiliations:** ^1^ Department of Oncology, Hangzhou Hospital of Traditional Chinese Medicine Affiliated to Zhejiang Chinese Medical University, Hangzhou, China; ^2^ Tongde Hospital of Zhejiang Province, Hangzhou, China; ^3^ Zhejiang Chinese Medical University, Hangzhou, China; ^4^ Department of Traditional Chinese Medicine, Ruijin Hospital, Shanghai Jiao Tong University, School of Medicine, Shanghai, China; ^5^ Department of Oncology, The First Affiliated Hospital of Zhejiang Chinese Medical University, Hangzhou, China

**Keywords:** renal cell carcinoma, cabozantinib, tyrosine kinase inhibitors, immune checkpoint inhibitors, adverse events

## Abstract

**Context:**

Cabozantinib combined with immune checkpoint inhibitors (ICIs) has brought a new therapeutic effect for the medical treatment of renal cell carcinoma (RCC).

**Objectives:**

We performed a meta-analysis of randomized controlled trials and single-arm trials to evaluate the efficacy and safety of cabozantinib plus ICIs in RCC.

**Methods:**

We extracted data from PubMed, Cochrane, Medline and Embase databases, and rated literature quality through Cochrane risk of bias tool and MINORS. RevMan5.3 software was used to analyze the results of randomized controlled trials and single-arm trials.

**Results:**

A total of 7 studies were included. Treatment with cabozantinib plus ICIs improved PFS [HR 0.75, (95%CI: 0.52, 1.08), *p* = 0.12] and the OS [HR 0.80, (95%CI: 0.60, 1.07), p = 0.13] in randomized controlled trials. Meanwhile, the result of the ORR in randomized controlled trials was [risk ratio (RR) 1.37, (95%CI: 1.21, 1.54), *p* < 0.00001] and in single-arm trials was [risk difference (RD) 0.49, (95%CI: 0.26, 0.71), *p* < 0.0001].

**Conclusion:**

Cabozantinib plus ICIs prolonged the PFS and OS, and improved ORR in patients with RCC. Our recommendation is to use cabozantinib plus ICIs to treat advanced RCC, and to continuous monitor and manage the drug-related adverse events.

**Systematic Review Registration:**

identifier CRD42023455878.

## 1 Introduction

On the basis of the Global Cancer Statistics 2020, there were 431,288 new cases and 179,368 death cases of kidney cancer in 185 countries. Moreover, the incidence of male was 1.69 times that of female (Sung et al., 2021). About 85% of patients with kidney cancer are renal cell carcinoma (RCC), which can be divided into the most common type of clear cell renal cell carcinoma (ccRCC), papillary renal cell carcinoma (pRCC), and chromophobe renal cell carcinoma (chRCC) with the lowest risk of metastasis (Kane et al., 2008; Smaldone et al., 2013; Motzer et al., 2023). According to data collected in the United States between 2010 and 2016, approximately one-third of RCC patients have had local or distant metastases at the time of diagnosis (meaning poor prognosis), and the 5-year survival rate for these patients was only 12% (Ljungberg et al., 2011; Motzer et al., 2023). Radical nephrectomy is still the gold standard therapy for the local renal mass in any patient who is not suited for nephron-sparing surgery. However, approximately 30% RCC relapse postoperatively (Dell Atti et al., 2022; Makhov et al., 2018). Abnormal angiogenesis is the hallmark of malignant tumor. The occurrence of RCC is based on abnormal angiogenesis, and the most common ccRCC is associated with altered signaling pathways such as von Hippel-Lindau (VHL), vascular endothelial growth factor receptor (VEGFR), and PI3K (phosphatidylinositol-3 kinase)/AKT/mammalian target of rapamycin (mTOR) (Banumathy and Cairns, 2010; Jonasch et al., 2014; Ganner et al., 2021). RCC is immune-infiltrated, characterized by high density infiltration of CD8^+^ T cells and high expression of programmed death ligand 1 (PD-L1), which illustrates the importance of the programmed death 1 (PD-1)/PD-L1 checkpoint in regulating RCC tumor growth (Griffiths et al., 2007; Vuong et al., 2019). Therefore, immune checkpoint inhibitors (ICIs) have been identified as another promising treatment option.

With the emergence of new anti-VEGF targeting drugs and ICIs, RCC patients have ushered in a new turning point. In first- and second-line treatments of systemic therapy, targeted therapy utilizing tyrosine kinase inhibitors (TKIs), and/or anti-vascular endothelial growth factor (VEGF) antibodies were widely used (Motzer et al., 2022a). Cabozantinib is an antiangiogenic inhibitor, also a TKI of multiple targets (Yakes et al., 2011). Because cabozantinib can improve PFS and OS of RCC patients, it was approved for the first-line treatment in low - and moderate-risk patients (Sammarco et al., 2023). However, compared with monotherapy, TKIs (especially cabozantinib) combined with ICIs have shown superior efficacy in first-line treatment over patients with advanced RCC (Albiges et al., 2021; Bedke et al., 2021). With the advantages of PFS and OS have shown in the CheckMate 9ER, cabozantinib in combination with nivolumab was approved for patients with previously untreated advanced RCC (Choueiri et al., 2021; Motzer et al., 2022b). Currently, the first-line treatment for RCC is a combination of TKIs (especially cabozantinib) and ICIs (Bedke et al., 2021; Navani et al., 2022). The purpose of this article is to explore the efficacy and drug-related adverse events of cabozantinib combined with ICIs in treating RCC. Seven trials with a total of 1965 patients are included in our review. We analyzed cabozantinib plus ICIs in treating RCC patients from the aspect of mechanism of action, combined benefits and clinical efficacy. Weighting the advantages and disadvantages of drugs to provide new ideas for readers in related fields and provide reference value for clinical treatment.

## 2 Methods

### 2.1 Search strategy

The research designer (JS) established a search formula based on subject terms and free terms provided by PubMed, and then conducted a literature search on PubMed, Cochrane Central, Embase and Medline databases. The deadline is 27 July 2023. In order to retrieve more articles, we set the Medical Subject Headings (MeSH) term as tumor and free term as cabozantinib. The search formula is as follows, and the search results of each database are shown in Supplementary Appendix S1. And we have registered in the PROSPERO (No.CRD42023455878).

(1) Patients: the MeSH term is Neoplasms, the free terms are (Neoplasm) OR (Malignant Neoplasm) OR (Malignancy) OR (Malignancies) OR (Tumor) OR (Malignant Tumor) OR (Cancer) OR (Malignant Cancer).
(2) Intervention: The free terms are (Cabozantinib) OR (Cabometyx) OR (Cometriq) OR (XL 184) OR (XL-184) OR (XL184 cpd) OR (BMS907351) OR (BMS 907351) OR (BMS-907351).

### 2.2 Study details

#### 2.2.1 Participants/patients

Patients with histological or cytological diagnosed renal cell malignancies. Furthermore, we specifically sought out patients who had a sample size of at least 10 people in each group. Additionally, patients with good liver and kidney function, as well as bone marrow hematopoietic function, were necessary in order to ensure that they could complete the drug therapy successfully.

#### 2.2.2 Research design

In order to maintain a high level of accuracy and reliability, we meticulously evaluated the quality of the included studies. The research methods employed in this study were mainly prospective in nature, encompassing both randomized controlled trials and single-arm trials. The single-arm trial, also referred to as a single-arm clinical trial, is a type of research study where a control group is not included, and only an experimental group is analyzed. We mainly compared the efficacy of cabozantinib combined with ICIs with other drugs in renal cell carcinoma. The drugs in the intervention group should be cabozantinib combined with ICIs, and the drugs in the control group should not include cabozantinib plus ICIs. We similarly emphasized the need to include cabozantinib in combination with ICIs in the trial arm of single-arm trials. Consequently, any single-arm trails involving medications other than cabozantinib and ICIs were not considered in this study.

#### 2.2.3 Outcome indicators

The main outcome that will be measured and analyzed in this study is progression-free survival (PFS). In addition to PFS, secondary outcome indicators such as overall survival (OS), complete response (CR), partial response (PR) will also be evaluated. Furthermore, the safety of the treatment will be evaluated by analyzing the most common types of drug-related adverse events experienced by the participants.

#### 2.2.4 Exclusion criteria

Animal and cell experiments, retrospective studies, and case reports were excluded. For studies with the same registration number, we selected the latest research results for analysis. Articles whose data could not be extracted and original authors could not be contacted were not included in the study. The intervention group should not include chemotherapeutics or other types of targeted drugs.

### 2.3 Data extraction

Two authors (YW and CN) scanned the titles, keywords and abstracts independently by using Endnote. For doubtful studies, they would read the full literature and then cross-check. When there was a controversial document, it was reviewed and confirmed by a third author (JZ) to finally determine the inclusion of the study. The extraction contents include: author’s name and publication year, study type, mean age and sample size in each group, drugs used in the intervention group or control group, primary and secondary outcomes, drug-related adverse events.

### 2.4 Assessment of risk of bias

Two individuals (YD and ZC) assessed the quality of randomized controlled trials by using the Cochrane risk bias tool and single-arm trials by using the Methodological Index for Non-randomized Studies (MINORS).

### 2.5 Data synthesis and analysis

We established a database of “Cabozantinib in combination with immune checkpoint inhibitors for renal cell carcinoma”. The quality of randomized controlled studies was assessed with reference to the Cochrane Quality Risk Assessment table, while the Minors score was used for single-arm experiments, both of them should be of high quality. Meta-analysis was performed by using RevMan 5.3. For outcomes of randomized controlled studies expressed as hazard ratios (HR) with 95% confidence intervals (CI) or relative risk (RR) with 95% CI. However, single-arm studies took rate as the outcome index, its incidence P and its standard error SE(P) can be calculated according to the following two methods:

(1) Method one

$$\text{The formula is }P=X/n;\text{ SE}\left(P\right)={\left(P\left(1‐P\right)/n\right)}^{\wedge }0.5$$

X is the number of occurrences of an event and n is the sample size.

Conditions of use: n is large enough, incidence P is not close to 0 and 1, both n*P and n*(1-p) are greater than 5, then the sampling distribution of P is close to normal distribution, and Risk Difference analysis is selected.

(2) Method two

When the n*P and n*(1-p) are not greater than 5 or the number of events is 0, that is, the incidence rate P does not meet the normal distribution, P and SE are calculated using the following method and the Odds Ratio is selected as follows:

$$\text{The formula is }P=\ln\left(\text{odds}\right)=\ln\left(X/\left(n–X\right)\right);\text{ SE}\left(P\right)=\text{SE}\left(\ln\left(\text{odds}\right)\right)=(1/X+1/\left(n–X\right){)}^{\wedge }0.5$$

To assess the presence of heterogeneity among the studies, two statistical variables were employed. The Cochran Q statistic of *p*-value and *I* ^*2*^ statistic was used. If the *I* ^*2*^ statistic was greater than 50% or the corresponding *p*-value was less than 0.10 (indicating a high level of heterogeneity), random effects models were employed to estimate the combined effect sizes of the studies. Random effects models take into account the heterogeneity between studies and assume that the true effect sizes vary across studies. Besides, sensitivity analysis or further subgroup analysis is required. On the other hand, if there was no significant heterogeneity (i.e., the *I* ^*2*^ statistic was less than 50% and the corresponding *P* -value was greater than 0.10), fixed effects models were applied.

## 3 Results

### 3.1 Search process

**FIGURE 1:**
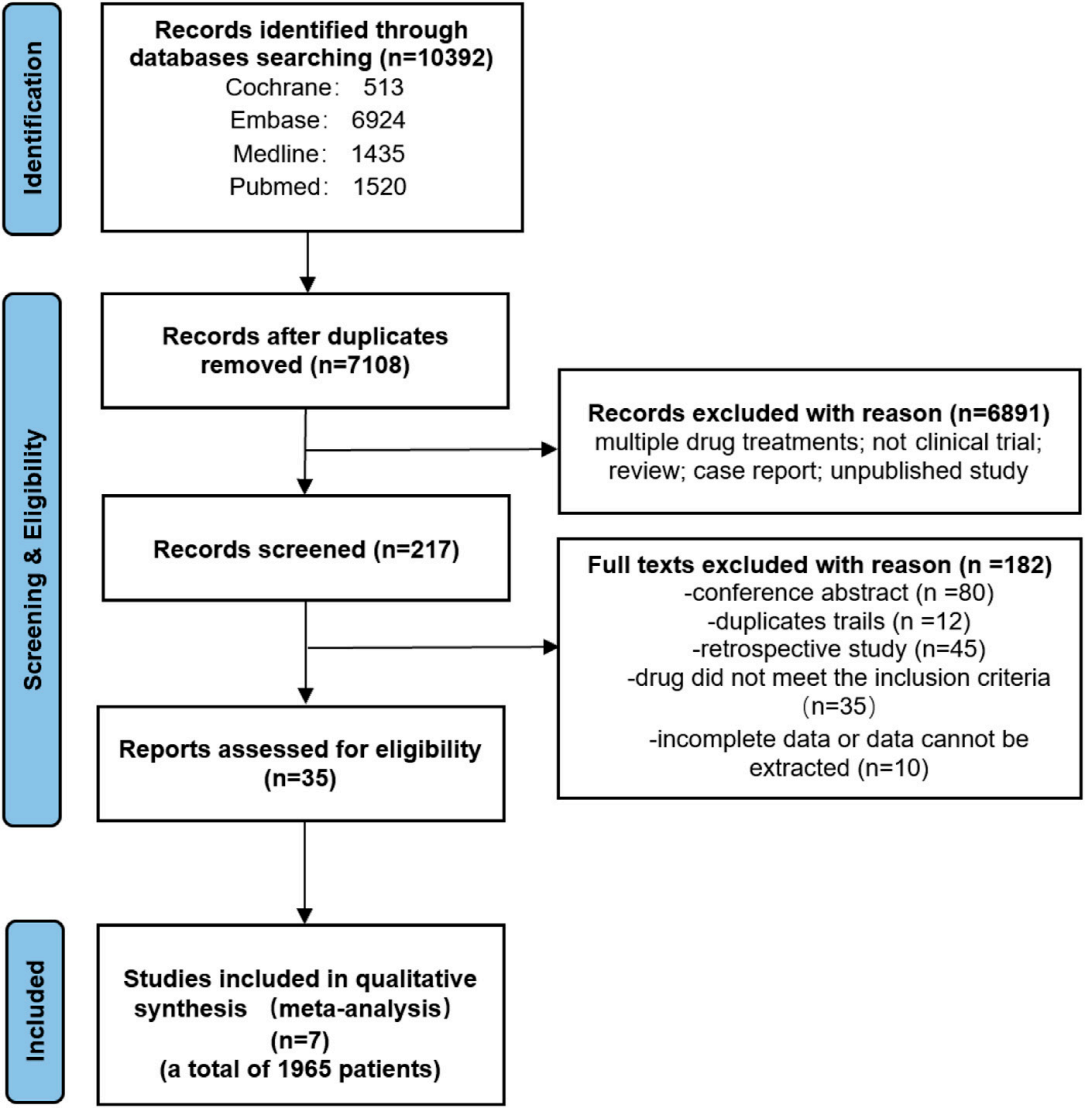
PRISMA flow diagram of search.

We searched the literature in four databases in accordance with search strategy to initially obtained 10,392 articles, removing 3,284 duplicate articles, remaining 7,108 articles. Then, we excluded 6,949 articles to further narrow down the list of articles, including non-clinical study, case report, unpublished study, review, non-cabozantinib plus immune checkpoint inhibitors, among others. Finally, we left 7 studies to analyse the study results, including 3 randomized controlled trials and 4 single-arm trials, the PRISMA flow diagram as shown in Figure 1.

### 3.2 Quality assessment

Three randomized controlled trials were all of high quality. Though two studies were open-lable trials, they were all mentioned the allocation concealment and had no outcome bias. Another 4 single-arm trials were also rated as high quality, and the Minors scores were all higher than 14 points with a full score of 16. The figure of quality evaluation of randomized controlled trials as illustrated in Figure 3 and the Minors score was demonstrated in Table 1.

**TABLE 1:** Overview of studies’ characteristics in randomized controlled trials and single-arm trials.

NO.	References	Study design	Sample size	Average age	Drugs	ORR	MINORS score
1	Motzer 2022	III	323/328	62(20-90)/61(28-86)	Niv + Cab/Sun	40/17CR; 140/76PR	—
2	Choueiri 2023	III	276/274	61(29-82)/60(28-85)	Cab + Niv + Ipi/Niv + Ipi + Pla	0/2CR; 105/102PR	—
3	Pal 2023	III	263/259	62(20-85)/63(18-89)	Ate + Cab/Cab	7/9CR; 112/89PR	—
4	Pal 2021	Ib	34	68 (39–87)	Ate + Cab	—	15
5	Pal 2021	Ib	36	60 (42–82)	Ate + Cab	—	15
6	Pal 2021	Ib	32	62 (37–78)	Ate + Cab	—	15
7	Apolo 2022	II	50	60 (40–84)	Niv + Cab + Ipi	4CR; 18PR	15
8	Lee 2022	III	40	57 (33–78)	Niv + Cab	19PR	14
9	Kessler 2023	I/II	45	61 (54–69)	Pem + Cab	1CR; 24PR	15

CR, complete response; PR, particial response; Cab, cabozantinib; Sun, sunitinib; Ipi, ipilimumab; Ate, atezolizumab; Pem, pembrolizumab; Niv, nivolumab; Pla, placebo.

### 3.3 Study characteristics

Three randomized controlled trials and 4 single-arm trials were all cabozantinib plus immune checkpoint inhibitors. We extracted basic information about each study, including author’s name, sample size, average age, drugs, and outcomes, as shown in Table 1.

### 3.4 Outcomes

#### 3.4.1 PFS and OS

**FIGURE 2:**
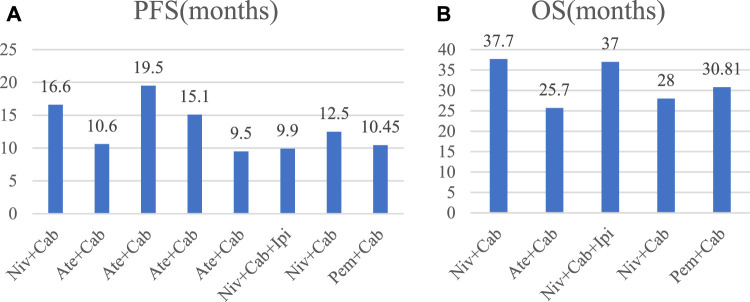
Bar chart of PFS and OS in each study. Cab, Cabozantinib; Ipi, ipilimumab; Ate, atezolizumab; Pem, pembrolizumab; Niv, nivolumab.

**FIGURE 3:**
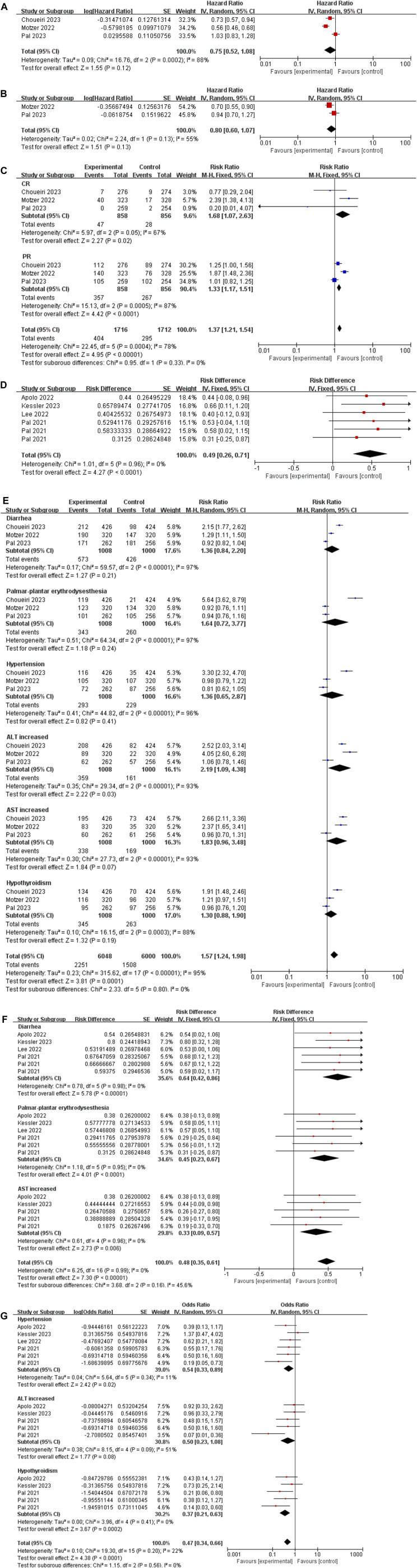
**(A)** Forest plot of the PFS in randomized controlled trials. **(B)** Forest plot of the OS in randomized controlled trials. **(C)** Forest plot of the ORR in randomized controlled trials. **(D)** Forest plot of the ORR in single-arm trials. **(E)** The six most common AEs in randomized controlled trials. **(F,G)** The six most common AEs in single-arm trials.

We extracted the values of PFS and OS of each study and converted them into intuitive graph, as shown in Figure 2. Since there was no control group in the single-arm trials, we only analyzed PFS and OS in the randomized controlled trials. The risk of death in RCC patients was 75% of the control group, and the median PFS was [HR 0.75, (95%CI: 0.52, 1.08), *p* = 0.12], as described in Figure 3A. While the OS was [HR 0.80, (95%CI: 0.60, 1.07), *p* = 0.13], as displayed in Figure 3B.

#### 3.4.2 ORR in randomized controlled trials

The result of the ORR was [risk ratio (RR) 1.37, (95%CI: 1.21, 1.54), *p* < 0.00001], among CR was [RR 1.68, (95%CI: 1.07, 2.63), *p* = 0.02], and PR was [RR 1.33, (95% CI: 1.17, 1.51), *p* < 0.0001], as depicted in Figure 3C.

#### 3.4.3 ORR in single-arm trials

The result of the ORR in single-arm trials was [risk difference (RD) 0.49, (95%CI: 0.26, 0.71), *p* < 0.0001], as depicted in Figure 3D.

#### 3.4.4 Adverse events

We extracted the six most common adverse events of cabozantinib plus immune checkpoint inhibitors in RCC patients. (1) In randomized controlled trials: Diarrhea: [RR 1.36, (95% CI: 0.84, 2.20), *p* = 0.21]; Palmar-plantar erythrodysesthesia: [RR 1.64, (95% CI: 0.72, 3.77), *p* = 0.24]; Hypertension: [RR 1.36, (95% CI: 0.65, 2.87), *p* = 0.41]; Alanine transaminase (ALT) increased: [RR 2.19, (95% CI: 1.09, 4.38), p = 0.03]; Aspartate transaminase (AST) increased: [RR 1.83, (95% CI: 0.96, 3.48), *p* = 0.07]; Hypothyroidism [RR 1.30, (95% CI: 0.88, 1.90), *p* = 0.19]. (2) In single-arm trials: Diarrhea: [RD 0.64, (95% CI: 0.42, 0.86), *p* < 0.00001]; Palmar-plantar erythrodysesthesia (PPE): [RD 0.45, (95% CI: 0.23, 0.67), *p* < 0.0001]; AST increased: [RD 0.33, (95% CI: 0.09, 0.57), p = 0.006]; AST increased: [OR 1.83, (95% CI: 0.96, 3.48), *p* = 0.07]; Hypertension: [OR 0.54, (95% CI: 0.33, 0.89), *p* = 0.02]; ALT increased [OR 0.50, (95% CI: 0.23, 1.08), *p* = 0.08]; Hypothyroidism [OR 0.37, (95% CI: 0.21, 0.63), *p* = 0.0002], as demonstrated in Figures 3E-G.

## 4 Discussion

Seven trials with a total of 1965 patients are included in our review (Pal et al., 2021a; Apolo et al., 2022; Motzer et al., 2022c; Lee et al., 2022; Choueiri et al., 2023; Kessler et al., 2023; Pal et al., 2023). The results of the summarized RCT showed high heterogeneity in PFS and OS, which may be related to the lack of significant difference in the study of atezolizumab plus cabozantinib versus cabozantinib conducted by Pal et al. (Pal et al., 2023). In addition to the PFS and OS, the data of ORR (CR and PR) by Pal et al. also demonstrated no significant difference when compared to the control group. Among the three randomized controlled trials, Motzer et al. (Motzer et al., 2022c) achieved the most favorable PFS, OS, and ORR with nivolumab plus cabozantinib in patients with previously untreated clear-cell advanced RCC. Choueiri et al. (Choueiri et al., 2023) included patients who had received a previous regimen in addition to PD-1/PD-L1 plus CTLA-4 inhibitors without TKIs and therefore remained statistically responsive to cabozantinib plus ICIs. Meanwhile, the study conducted by Pal et al. (Pal et al., 2023) included up to 50% of patients who had previously received TKIs, and almost all patients had received ICIs as a second-line therapy. Therefore, the results of Pal’s study indicated that there was no significant difference between the combination of cabozantinib and atezolizumab and the use of cabozantinib alone (Pal et al., 2023). Furthermore, the study concluded that there was no clinical benefit in continuing the use of a PD-L1 inhibitor in patients with checkpoint inhibitor-resistant RCC who were already receiving TKI therapy. This finding helps explain the high heterogeneity or statistical insignificance (*p* > 0.05) observed in the PFS, OS and ORR outcomes of our study. Due to the small number of included studies, it is important to acknowledge that the available evidence is limited. Despite this, all the indicators in the various studies generally demonstrated positive outcomes. However, it is crucial to highlight that the results also exhibited a significant degree of variation. Therefore, when performing statistical analysis, it is also the case that statistical bias due to the influence of a single study leads to high heterogeneity or observe a lack of statistical significance solely. Besides, due to the different types of ICIs and small sample sizes, there might also be some bias in our study.

The results of a single arm trial demonstrated that the combined use of cabozantinib and ICIs can significantly improve the ORR in patients with RCC. This finding further supports the recommendation for early utilization of cabozantinib in combination with ICIs for the treatment of advanced RCC patients. Cabozantinib alone has been shown to be more effective in the treatment of RCC, provided a median PFS superior to sunitinib in moderate-to-low risk patients (Lalani et al., 2019) and improved PFS and ORR in patients by reducing mortality by 20% (Choueiri et al., 2017). A meta-analysis also showed that cabozantinib as a follow-up first-line therapy had a longer likelihood of OS and PFS compared to everolimus, axitinib, sorafenib, ect (Amzal et al., 2017). In one randomized controlled trial, the median OS was 21.4 months with cabozantinib and 16.5 monthswith everolimus (*p* = 0.0008) and cabozantinib extended the time to deterioration (Choueiri et al., 2016; Cella et al., 2018). Another study compared cabozantinib to sunitinib, and it was found that patients treated with cabozantinib had significantly longer OS (26.6 months) compared to those treated with sunitinib (21.2 months) (Choueiri et al., 2018). This suggests that cabozantinib as a single-agent treatment has shown remarkable efficacy in improving patient outcomes. When cabozantinib was combined with ICIs, it was found to further enhance patient outcomes. The combination therapy improved PFS and OS compared to cabozantinib alone. In particular, the PFS was significantly prolonged for patients treated with cabozantinib combined with nivolumab (16.6 months) compared to those treated with sunitinib monotherapy (8.3 months) (Motzer et al., 2022c). Patients continue to report demonstrate treatment with nivolumab plus cabozantinib versus sunitinib reduced the risk of meaningful deterioration in health-related quality of life scores and showed a decreased risk of being bothered by treatment side-effects (Motzer et al., 2022c). These findings highlight the importance of cabozantinib as a TKI and underscore the necessity of ICIs in reducing immune escape.

**FIGURE 4:**
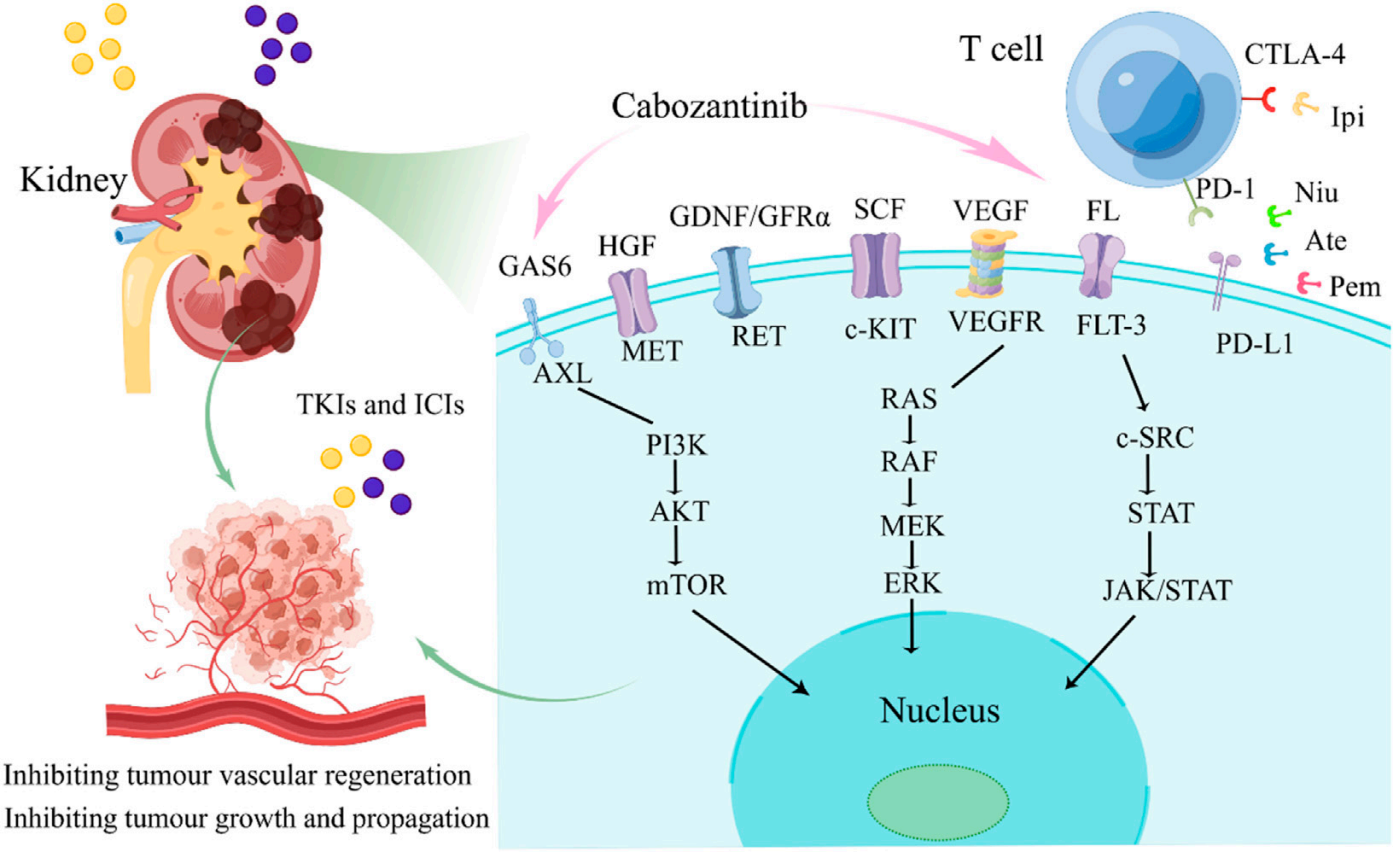
The major signaling pathways of cabozantinib. FL, FLT-3 ligand; FLT-3, FMS-like tyrosine kinase-3; GDNF, glial cell-derived neurotrophic factor; GFRα, GDNF family receptor α; RET, rearranged during transfection; HGF, hepatocyte growth factor; MET, mesenchymal-epithelial transition factor; SCF, stem cell factor; c-KIT, proto-oncogene proteins c-kit; VEGF, vascular endothelial growth factor; VEGFR, vascular endothelial growth factor receptor; GAS6, Growth Arrest Specific Protein 6; AXL, AXL receptor tyrosine kinase; TKIS, tyrosine kinase inhibitors; ICIs, immune checkpoint inhibitors; Ipi, ipilimumab; Ate, atezolizumab; Pem, pembrolizumab; Niv, nivolumab.

Von-Hippel Lindau/Hypoxia-Inducible Factor (VHL/HIF) promotes VEGFR activation and expression, inhibits apoptosis and stimulates tumor progression through the PI3K/AKT/mTOR pathway (Linehan et al., 2009; Banumathy and Cairns, 2010; Yeo et al., 2017). In addition, the tyrosine kinase receptor MET is induced by hepatocyte growth factor (HGF) to activate the PI3K/AKT/mTOR and RAS/RAF/MAPK pathways, thereby promoting the growth and metastasis of renal cancer cells (Eder et al., 2009; Alonso-Gordoa et al., 2019). Meanwhile, MET is associated with resistance mechanisms to targeted therapies, including EGFR and VEGFR inhibitors (Xie et al., 2016). Cabozantinib acts on all the targets and pathways mentioned above, including MET, VEGFR-2, RET, AXL, and FLT-3, mainly inhibits angiogenesis, tumor cell growth and propagation through rearranged during transfection (RET)/RAS/RAF/mitogen-activated protein kinase (MAPK), PI3K/AKT/mTOR, and janus kinase (JAK)/signal transducer and transcription activator (STAT) (Su et al., 2022; Su et al., 2023), as displayed in Figure 4. Cabozantinib also has immunomodulatory properties, which shift the tumor microenvironment from immunosuppressive to immunopermissive (Kwilas et al., 2014). The antivascular mechanism of TKIs determines the normalization of tumor vascular structure and increases the infiltration of immune cells by inhibiting tumor-related angiogenesis (Huang et al., 2013). These drugs reduce the differentiation of cells with immunosuppressive functions, such as promoting the differentiation of monocytes into mature dendritic cells, restricting the differentiation of macrophages into M2 types with immunosuppressive activities, thereby restoring immunosensitive tumor microenvironment (TME) and enhancing the effect of ICIs (Osada et al., 2008; Movahedi et al., 2010).

**FIGURE 5:**
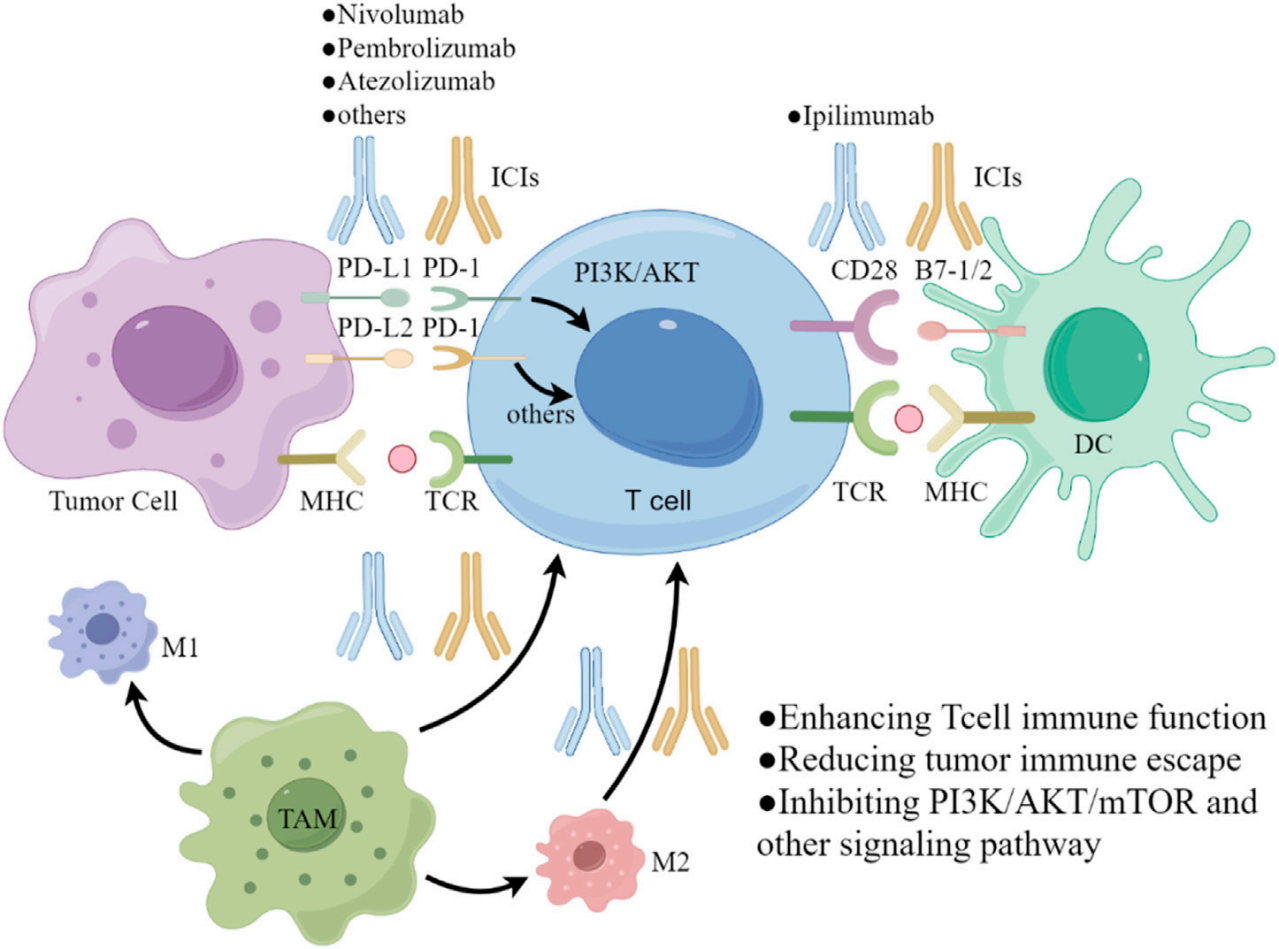
The major signaling pathways of immune checkpoint inhibitors.

RCC is highly immunogenic and PD-L1 is widely expressed in RCC, which illustrates the importance of the PD-1/PD-L1 checkpoint in regulating RCC tumor growth (Michael and Pandha, 2003; Griffiths et al., 2007). The interactions of overexpression of PDL1 and PD-1 receptor leads to T cell downregulation and impotence, downregulating the host immune response to RCC. ICIs include PD-L1 inhibitors (such as atezolizumab, pembrolizumab, nivolumab), and cytotoxic T lymphocyte antigen 4 (CTLA-4) inhibitors (like ipilimumab) that promote a durable host immune response against tumor growth by inhibiting tumor-induced downregulation of host T cells (Parry et al., 2005; Thompson et al., 2005; Griffiths et al., 2007). The blinding process of PD-1/PD-L1 that can be summarized in three aspects: recruiting immunosuppressive cells, reducing immunogenicity, and evading immune surveillance (Dong et al., 2002; Schreiber et al., 2011; French et al., 2017). Inhibition of PD-1/PD-L1 pathway can not only restore anti-cancer immunity, but also can directly promote hypoxia and apoptosis of tumor cells, restricting the growth of them by inhibiting MAPK signaling pathway (Liotti et al., 2021). Tumor-associated macrophage (TAM) can be divided into alternately activated macrophages (M2) and classically activated macrophages (M1), in which M2 plays a dominant role, and inhibits anti-tumor function T cells and B cells by expressing inhibitory ligands of PD-L1/L2 and CD80/86 (Sica et al., 2006; Butte et al., 2007; Bloch et al., 2013). TAM upregulates PD-1-related genes, which in turn promotes the binding of PD-1 and PD-L1. This binding leads to the phosphorylation of tyrosine residues, which then bind to protein tyrosine phosphatases and activate triggering downstream pathways such as PI3K/AKT. These pathways ultimately inhibit T cell signaling and promote T cell depletion, which negatively affects the immune response (Dong et al., 2002; Chevrier et al., 2017). Additionally, TAM has a direct impact on the expression of PD-1 on CD8^+^ T cells and PD-L1 on tumor cells. By doing so, TAM can regulate the activation and proliferation of macrophages through the PI3K/AKT/mTOR signaling pathway (Hartley et al., 2018). In this way, TAM can negatively regulate the immune response. However, PD-1/PD-L1 inhibitors can effectively block the tumor-promoting effects induced by TAM. These inhibitors enhance the immune activity of effector T cells, which are responsible for killing tumor cells to limit the progression of tumor invasion. These inhibitors can also work in coordination with other immune checkpoint inhibitors (ICIs) to further enhance the immune response and limit tumor growth and propagation (Fiegle et al., 2019; Xiong et al., 2019), as demonstrated in Figure 5. VEGF has an immunosuppressive effect, allowing Tregs and the accumulation of myeloid suppressor cells (Rizzo et al., 2022). Therefore, inhibition of angiogenesis can have a significant impact on the immunosuppressed tumor microenvironment, thereby inhibiting TAM, making combination with ICIs a powerful anti-tumor strategy for treating advanced RCC (Kasherman et al., 2022). A phase Ⅲ study of nivolumab plus cabozantinib versus sunitinib, the combined group had a 49% lower risk of disease progression or death compared to the control group, and the median PFS in the combined group was twice that of sunitinib (16.6 months vs. 8.3 months) (Motzer et al., 2022c). Based on these results, cabozantinib in combination with nivolumab was approved by the Food and Drug Administration (FDA) in January 2021 for first-line therapy in patients with advanced ccRCC. Multiple clinical trials of TKIs in combination with ICIs demonstrated higher response rates and improved survival outcomes, which supported by the latest National Comprehensive Cancer Network (NCCN) and European Association of Urology (EAU) 2021 guidelines (Pal et al., 2021a; Tung and Sahu, 2021; Apolo et al., 2022; Motzer et al., 2022c; Lee et al., 2022; Choueiri et al., 2023; Kessler et al., 2023; Pal et al., 2023).

**FIGURE 6:**
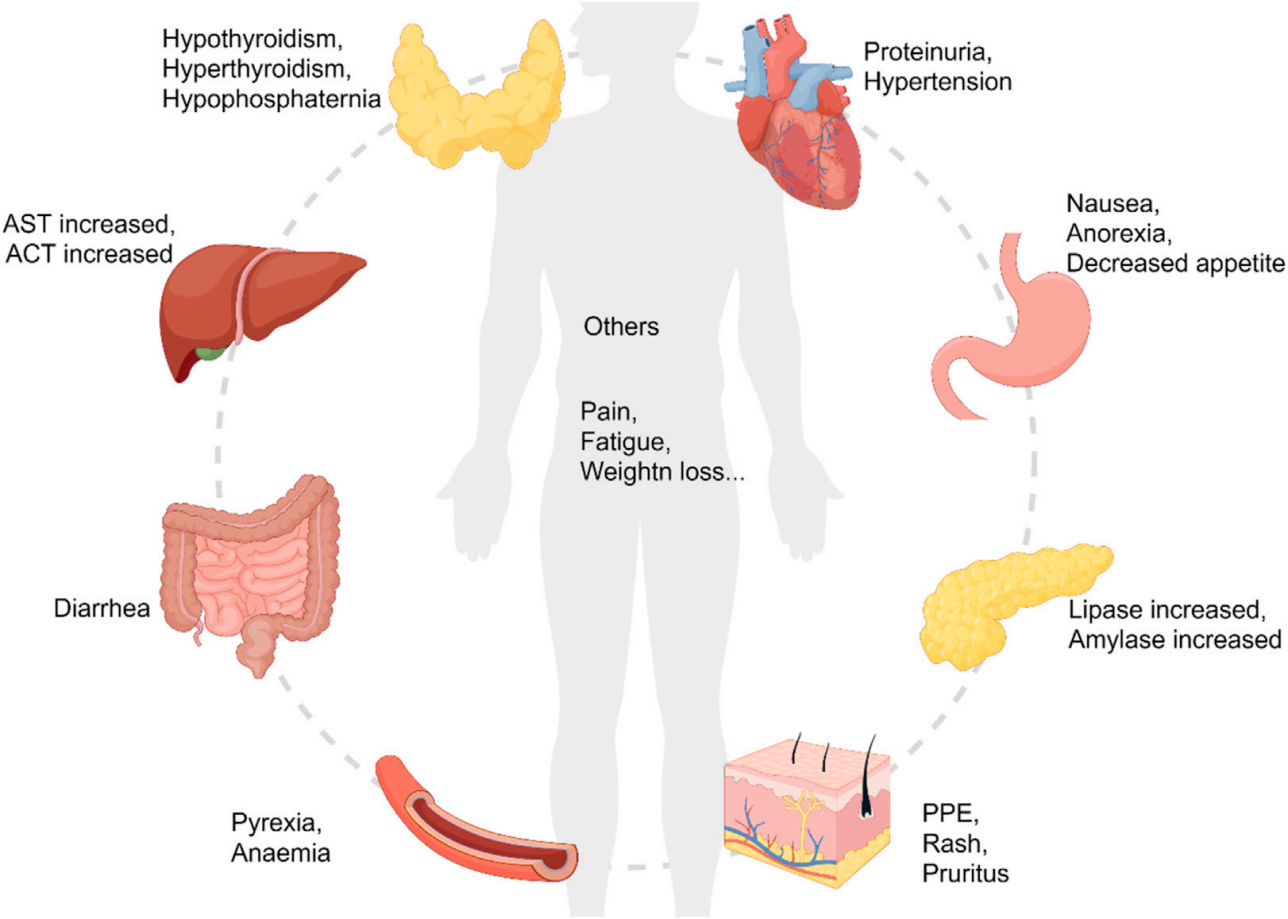
The common adverse events of cabozantinib plus immune checkpoint inhibitors.

We presented the common AEs of cabozantinib combined with ICIs to readers through graphical and literal models, as shown in Figure 6. After statistical analysis, we extracted six common AEs, including diarrhea, palmar-plantar erythrodysesthesia, hypertension, ALT increased, AST increased, hypothyroidism. In randomized controlled trials, we have extracted the number of patients of adverse drug reactions in both the experimental group and the control group. And the number of patients of adverse drug reactions in the single-arm trial was only extracted in the experimental group. By extracting drug-related adverse events of any severity from each individual study, it enabled us to conduct a meta-analysis that would provide a robust evaluation of the overall safety of the drugs under investigation. From the results of the AEs, although the results of AEs in randomized controlled trials were high heterogeneous and not statistically significant, considering that the control group was also targeted drugs or ICIs, it was a reasonable bias. From a summary of six common AEs, cabozantinib combined with ICIs had higher AEs rate than either targeted agents or ICIs alone [RR 1.57, (95% CI: 1.24, 1.98), *p* = 0.0001] in the randomized controlled trials. According to the methodology, AEs in the single-arm test were analyzed in two subgroups, with statistical differences in each group [RD 0.48, (95%CI: 0.35, 0.61), *p* < 0.00001], [OR 0.47, (95%CI: 0.34, 0.66), p < 0.0001]. Common AEs in cabozantinib usually include diarrhea, hypertension, abnormal liver function tests, and PPE. These adverse effects have also been observed with other anti-angiogenic inhibitors in patients with RCC. Continuous monitoring of blood pressure, antidiarrheal treatment and other symptomatic treatments can reduce the incidence of adverse reactions. The AEs observed at sunitinib were generally similar to cabozantinib, but with a lower incidence of PPE, anorexia, and weight loss, and with a higher incidence of hematological toxicities such as thrombocytopenia and neutropenia (Eisen et al., 2012; Choueiri et al., 2015; Choueiri et al., 2016). While ICIs can cause a variety of endocrine toxicity, including thyroid dysfunction, adrenal insufficiency, type 1 diabetes, etc. (Antonelli et al., 2018). Using TKIs in combination with ICIs was associated with a higher risk of full-grade and grade 3-4 diarrhea, AST/ALT increased or hypothyroidism in all grades (Rizzo et al., 2022).

Cabozantinib undergoes metabolism in the liver through an enzyme called CYP3A4 with a relatively long half-life of approximately 99 h. When taking cabozantinib, it should be avoided with high-fat foods to increase the concentration of the drug in the bloodstream, leading to higher drug exposure. To prevent this, it is recommended to take cabozantinib at least 1 h before or 2 h after eating (McGregor et al., 2022). Typically, cabozantinib-associated AEs occur within weeks of the start of treatment, and the risk of AEs increases as the concentration of the drug increases or clearance decreases. Hence, dose adjustment is a commonly used strategy in administering cabozantinib to manage AEs (McGregor et al., 2022). A Phase 1b study (COSMIC-021) evaluated 40 mg and 60 mg doses of cabozantinib in combination with atezolizumab in patients with RCC. Results showed that the 60 mg group had a higher incidence of AEs, such as PPE (29% versus 56%), decreased appetite (24% versus 53%), and proteinuria (6% versus 22%). Meanwhile, the dose reduction rate was higher in the 60 mg group (56% vs. 86%) (Pal et al., 2021b). For intolerable AEs, cabozantinib can be reduced from 40 mg/day to 20 mg/day treatment or even discontinued (Tran et al., 2023). Because ICIs does usually not allow dose reduction, grade 3 immune-related adverse events (irAEs) or some grade 2 irAEs are often treated with dose maintenance and immunosuppression (McGregor et al., 2022). IrAEs are managed with oral or intravenous immunosuppressants such as prednisolone or methylprednisolone. Depending on the severity of irAEs, the dose is 0.5–2 mg/kg/day or equivalent and is most commonly used with a reduction of at least 4 weeks (Haanen et al., 2018; Brahmer et al., 2021; Schneider et al., 2021; Thompson et al., 2022). However, in cases of serious adverse reactions, such as recurrent grade 3 irAEs and ≥ grade 2 myocarditis requiring systemic immunosuppressive therapy, ICIs should be permanently discontinued (McGregor et al., 2022). Rashes of ≤ grade 3, topical corticosteroids are appropriate, but grade >3 rashes should be treated with systemic corticosteroids (equivalent to 0.5–1 mg/kg/day of predtisone) (Haanen et al., 2018; Brahmer et al., 2021; Schneider et al., 2021; Thompson et al., 2022). For pruritus, grade 1/2 should be treated with topical corticosteroids or antihistamines, and oral antihistamines should be added if pruritus remains uncontrolled (Wu and Lacouture, 2018; Phillips et al., 2019). Physical moisturizing, reducing skin friction on hands and feet is suitable for PPE patients (Su et al., 2023). Patients with fatigue should be evaluated for endocrine function, and supply corresponding hormone if endocrine disorders exist. Monitoring thyroid stimulating hormone (TSH) levels before and after treatment, using levothyroxine replace thyroid hormone in cases of hypothyroidism, while using antithyroid drugs inhibit in cases of hyperthyroidism (Ross et al., 2016). For ≥ grade 2 adrenal insufficiency, hormone replacement therapy should be selected (McGregor et al., 2022). Both cabozantinib and ICIs can cause liver enzymes elevated, ALT/AST and bilirubin should be monitored throughout treatment. For patients with AST/ALT >3 to ≤10 times the upper limit of normal (ULN) and bilirubin <2×ULN, corticosteroids may be considered (McGregor et al., 2022). Hypertension and proteinuria were also common AEs. By monitoring 24 h blood pressure and urine protein value, timely adjustment of drug dose is carried out (Su et al., 2023).

Our study is the first to comprehensively evaluate the efficacy and adverse events of cabozantinib combined with ICIs in patients with RCC. We believe that TKIs combined with ICIs in treating inoperable RCC patients is the current and future trend, especially cabozantinib plus ICIs. To personalize treatment, monitoring of drug efficacy biomarkers and AE risk related predictors is recommended for each patient. Prediction of tumor mutation burden, cytotoxic CD8 + T cells, VHL, cytokine, gene expression profile, and PD-L1 expression is beneficial to evaluate prognosis (Martin et al., 2023). A retrospective study found that age ≥60 years, GFR< 30 mL/min/1.73 m^2^, and single metastatic site were important predictors of VEGF targeted therapy discontinuation (McGregor et al., 2022). At present, there are still a number of clinical trials of TKIs combined with ICIs under study, and further studies are needed to distinguish the safety of various TKIs-ICIs combinations. We have listed the clinical trials meeting the requirements by searching the website of https://www.clinicaltrials.gov, as shown in Table 2, so that readers can consult and learn and provide information for clinical decision-making. While blindly pursuing the use of drugs to prolong PFS and OS patients, how to do a good job in adverse event management is particularly important. Our article has some limitations. Besides the bias caused by high heterogeneity or lack of statistical difference in some of the mentioned results earlier, there are a few other factors that should be considered. Firstly, the results of our study are relatively new, and we only examined clinical trials that were published in English. This choice of language may introduce a language bias, as valuable data from trials published in other languages could have been missed. Secondly, the data related to PFS and OS were relatively limited in our analysis. Furthermore, it is important to note that there are various types of ICIs available in the market. In our analysis, the three randomized controlled trials used different control drugs, which introduces a slight bias. To address these limitations and provide more comprehensive and accurate conclusions, it is crucial to conduct more clinical trials. More clinical trials are needed to determine the most appropriate ICIs with cabozantinib.

**TABLE 2:** Clinical studies of TKIs combined with ICIs in treating RCC patients registered in clinical trials.

NO.	Study design	TKIs	ICIs	Research number
1	I	Axitinib	Avelumab, Pembrolizumab	NCT04682587
2	I	Lenvatinib	Pembrolizumab	NCT05733715
3	II	Lenvatinib	Pembrolizumab	NCT04704219
4	I/II	Axitinib	Nivolumab	NCT03172754
5	II	Lenvatinib	Pembrolizumab	NCT04267120
6	I	Cabozantinib	Avelumab	NCT03200587
7	I/II	Lenvatinib	QL1706	NCT05262413
8	II	Axitinib	Pembrolizumab	NCT04995016
9	I/II	Cabozantinib	Pembrolizumab	NCT03149822
10	I	Sitravatinib	Nivolumab, Ipilimumab	NCT04518046
11	III	XL092, Sunitinib	Nivolumab	NCT05678673
12	II	Cabozantinib	Nivolumab, Ipilimumab	NCT05048212
13	—	Axitinib	Avelumab	NCT05650164
14	—	Axitinib	Avelumab	NCT05012865
15	—	Cabozantinib	Nivolumab	NCT04322955
16	III	Axitinib, Sunitinib	Avelumab	NCT02684006
17	—	Axitinib	Avelumab	NCT05394493
18	I	Axitinib	Avelumab	NCT02493751
19	II	Lenvatinib	Tislelizumab	NCT05877820
20	II	Axitinib	Toripalimab	NCT04385654
21	II	Axitinib	Nivolumab	NCT03595124
22	II	Axitinib	Avelumab	NCT05327686
		Cabozantinib	Ipilimumab, Nivolumab	
		Lenvatinib	Pembrolizumab	
23	I/II	Ibrutinib	Nivolumab	NCT02899078
24	II	Cabozantinib	Nivolumab	NCT03635892
25	II	Axitinib	Pembrolizumab	NCT04370509
26	II	Axitinib	Avelumab	NCT03341845
27	II	Lenvatinib	Pembrolizumab	NCT04955743
28	II	Axitinib	Pembrolizumab	NCT05263609
29	II	Axitinib	Nivolumab	NCT05817903
30	III	Cabozantinib	Atezolizumab	NCT04338269
31	I	Cabozantinib	Nivolumab	NCT05122546
32	—	Axitinib	Pembrolizumab	NCT05287464
33	II	Lenvatinib	Pembrolizumab	NCT05485896
34	II	Lenvatinib	Tislelizumab	NCT05485883
35	II	Cabozantinib	Nivolumab, Ipilimumab	NCT04413123
36	II	Axitinib	Tislelizumab	NCT05172440
37	I	XL092	Atezolizumab, Avelumab	NCT0385166

## 5 Conclusion and future directions

RCC usually has abnormal blood vessels and high concentration of immune infiltration. Cabozantinib combined with ICIs to inhibit VEGFR and activate immune cells to reduce the immune escape of tumor cells is currently a commonly used therapeutic combination. Cabozantinib can prolong PFS and OS more than sunitinib, and cabozantinib plus ICIs has better efficacy. Our subgroup analysis of PFS from randomized controlled trials showed that patients <65 years old, male gender, sarcomatoid features, absence of liver and bone metastases, and PD-L1 expression ≥1% were associated with better PFS, as detailed in Supplementary Appendix S2. Of course, the results of more large clinical trials are still needed to further confirm. However, while the combination of drugs brings significant efficacy, the incidence of adverse events also increases. In order to optimize treatment and improve patients’ quality of life, the management of adverse events is particularly important. The pain of patients can be alleviated through early monitoring of major organ indexes, early intervention of adverse events, and adjustment of medication before serious adverse events occur.

## Data Availability

The original contributions presented in the study are included in the article/Supplementary Material, further inquiries can be directed to the corresponding author.
