# Supplementary material for: Cabozantinib in combination with immune checkpoint inhibitors for renal cell carcinoma: a systematic review and meta-analysis
Source: Front Pharmacol. 2024 Apr 17;15:1322473. doi: 10.3389/fphar.2024.1322473 (PMC11061414; doi:10.3389/fphar.2024.1322473)
Supplement: Supplementary file 1 [file DataSheet1.docx]

**PICOS**

**P:**

**MeSH terms : Neoplasms**

**Free terms :**

(Tumor) OR (Neoplasm) OR (Cancer) OR (Malignant Neoplasm) OR (Malignancy) OR (Malignancies) OR (Malignant Tumor) OR (Malignant Cancer)

**I:** (Cabozantinib) OR (Cometriq) OR (Cabometyx) OR (XL 184) OR (XL184 cpd) OR (XL-184) OR (BMS 907351) OR (BMS907351) OR (BMS-907351)

**C:**

**O:**

**S:**

| **Database** | **Retrieval strategy** | **Number** |
| --- | --- | --- |
| **Pubmed** | #1 "Neoplasms "[Mesh] 3855744  (Tumor) OR (Neoplasm) OR (Cancer) OR (Malignant Neoplasm) OR (Malignancy) OR (Malignancies) OR (Benign Neoplasm) OR (Malignant Tumor) OR (Malignant Cancer)  #2 (Tumor):[Title/Abstract]  #3 (Neoplasm):[Title/Abstract]  #4 (Cancer):[Title/Abstract]  #5 (Malignant Neoplasm):[Title/Abstract]  #6 (Malignancy):[Title/Abstract]  #7 (Malignancies):[Title/Abstract]  #8 (Malignant Tumor):[Title/Abstract]  #9 (Malignant Cancer):[Title/Abstract]  #10 #1 OR #2 OR #3 OR #4 OR #5 OR #6 OR #7 OR #8 OR #9 5595145  #11 (Cabozantinib):[Title/Abstract]  #12 (Cometriq):[Title/Abstract]  #13 (Cabometyx):[Title/Abstract]  #14 (XL 184):[Title/Abstract]  #15 (XL184 cpd):[Title/Abstract]  #16 (XL-184):[Title/Abstract]  #17 (BMS 907351):[Title/Abstract]  #18 (BMS907351):[Title/Abstract]  #19 (BMS-907351):[Title/Abstract]  #20 #11 OR #12 OR #13 OR #14 OR #15 OR #16 OR #17 OR #18 OR #19  1618  #21 #10 AND #20 1520 | 1520 |
| **Cochrane Library** | #1 MeSH descriptor: [Neoplasms] explode all trees 111738  #2 (Tumor):ti,ab,kw  #3 (Neoplasm):ti,ab,kw  #4 (Cancer):ti,ab,kw  #5 (Malignant Neoplasm):ti,ab,kw  #6 (Malignancy):ti,ab,kw  #7 (Malignancies):ti,ab,kw  #8 (Malignant Tumor):ti,ab,kw  #9 (Malignant Cancer):ti,ab,kw  #10 #1 OR #2 OR #3 OR #4 OR #5 OR #6 OR #7 OR #8 OR #9 272790  #11 (Cabozantinib):ti,ab,kw  #12 (Cometriq):ti,ab,kw  #13 (Cabometyx):ti,ab,kw  #14 (XL 184):ti,ab,kw  #15 (XL184 cpd):ti,ab,kw  #16 (XL-184):ti,ab,kw  #17 (BMS 907351):ti,ab,kw  #18 (BMS907351):ti,ab,kw  #19 (BMS-907351):ti,ab,kw  #20 #11 OR #12 OR #13 OR #14 OR #15 OR #16 OR #17 OR #18 OR #19  587  #21 #10 AND #20 513 | 513 |
| **Medline** | #1 Neoplasms:MeSH Heading 3828107  #2 (Tumor):topic  #3 (Neoplasm):topic  #4 (Cancer):topic  #5 (Malignant Neoplasm):topic  #6 (Malignancy):topic  #7 (Malignancies):topic  #8 (Malignant Tumor):topic  #9 (Malignant Cancer):topic  #10 #1 OR #2 OR #3 OR #4 OR #5 OR #6 OR #7 OR #8 OR #9 5120889  #11 (Cabozantinib):topic  #12 (Cometriq):topic  #13 (Cabometyx):topic  #14 (XL 184):topic  #15 (XL184 cpd):topic  #16 (XL-184):topic  #17 (BMS 907351):topic  #18 (BMS907351):topic  #19 (BMS-907351):topic  #20 #11 OR #12 OR #13 OR #14 OR #15 OR #16 OR #17 OR #18 OR #19  1610  #21 #10 AND #20 1435 | 1435 |
| **EMBASE** | #1 'Neoplasms '/exp 6032887  #2 (Tumor):ab,ti  #3 (Neoplasm):ab,ti  #4 (Cancer):ab,ti  #5 (Malignant Neoplasm):ab,ti  #6 (Malignancy):ab,ti  #7 (Malignancies):ab,ti  #8 (Malignant Tumor):ab,ti  #9 (Malignant Cancer):ab,ti  #10 #1 OR #2 OR #3 OR #4 OR #5 OR #6 OR #7 OR #8 OR #9 7820333  #11 (Cabozantinib):ab,ti  #12 (Cometriq):ab,ti  #13 (Cabometyx):ab,ti  #14 (XL 184):ab,ti  #15 (XL184 cpd):ab,ti  #16 (XL-184):ab,ti  #17 (BMS 907351):ab,ti  #18 (BMS907351):ab,ti  #19 (BMS-907351):ab,ti  #20 #11 OR #12 OR #13 OR #14 OR #15 OR #16 OR #17 OR #18 OR #19  7185  #21 #10 AND #20 6924 | 6924 |
