# Supplementary material for: Cabozantinib in combination with immune checkpoint inhibitors for renal cell carcinoma: a systematic review and meta-analysis
Source: Front Pharmacol. 2024 Apr 17;15:1322473. doi: 10.3389/fphar.2024.1322473 (PMC11061414; doi:10.3389/fphar.2024.1322473)
Supplement: Supplementary file 2 [file Image1.pdf]

Study or Subgroup	log[Hazard Ratio]	SE	Weight	Hazard Ratio IV, Random, 95% CI
**1.1.1 ≥65 years old**				
Choueiri 2023	-0.21072103	0.2168341	13.9%	0.81 [0.53, 1.24]
Motzer 2022	-0.35667494	0.1613578	16.7%	0.70 [0.51, 0.96]
Pal 2023	0.05826891	0.171738	16.2%	1.06 [0.76, 1.48]
**Subtotal (95% CI)**			**46.7%**	**0.84 [0.65, 1.09]**
Heterogeneity: Tau² = 0.02; Chi² = 3.14, df = 2 (P = 0.21); I² = 36%				
Test for overall effect: Z = 1.30 (P = 0.19)				
**1.1.2 <65 years old**				
Choueiri 2023	-0.38566248	0.15831033	16.9%	0.68 [0.50, 0.93]
Motzer 2022	-0.69314718	0.12234007	18.7%	0.50 [0.39, 0.64]
Pal 2023	0.0295588	0.14182297	17.7%	1.03 [0.78, 1.36]
**Subtotal (95% CI)**			**53.3%**	**0.70 [0.46, 1.08]**
Heterogeneity: Tau² = 0.13; Chi² = 14.89, df = 2 (P = 0.0006); I² = 87%				
Test for overall effect: Z = 1.60 (P = 0.11)				
**Total (95% CI)**			**100.0%**	**0.76 [0.59, 0.99]**
Heterogeneity: Tau² = 0.08; Chi² = 20.66, df = 5 (P = 0.0009); I² = 76%				
Test for overall effect: Z = 2.05 (P = 0.04)				
Test for subgroup differences: Chi² = 0.51, df = 1 (P = 0.48), I² = 0%				

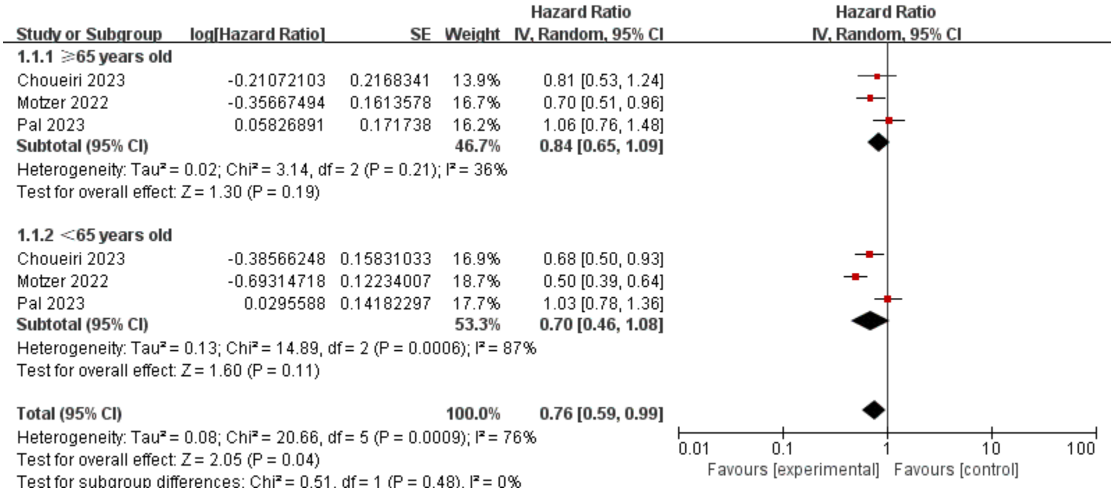

Study or Subgroup	log[Hazard Ratio]	SE	Weight	Hazard Ratio IV, Random, 95% CI
**1.2.1 Male**				
Choueiri 2023	-0.32850407	0.14677657	18.1%	0.72 [0.54, 0.96]
Motzer 2022	-0.65392647	0.11140756	19.8%	0.52 [0.42, 0.65]
Pal 2023	-0.02020271	0.12563176	19.1%	0.98 [0.77, 1.25]
**Subtotal (95% CI)**			**57.1%**	**0.71 [0.49, 1.05]**
Heterogeneity: Tau² = 0.10; Chi² = 14.32, df = 2 (P = 0.0008); I² = 86%				
Test for overall effect: Z = 1.72 (P = 0.09)				
**1.2.2 Female**				
Choueiri 2023	-0.30110509	0.25232859	13.1%	0.74 [0.45, 1.21]
Motzer 2022	-0.40047757	0.19553022	15.7%	0.67 [0.46, 0.98]
Pal 2023	0.29266961	0.22774496	14.2%	1.34 [0.86, 2.09]
**Subtotal (95% CI)**			**42.9%**	**0.87 [0.56, 1.34]**
Heterogeneity: Tau² = 0.09; Chi² = 5.78, df = 2 (P = 0.06); I² = 65%				
Test for overall effect: Z = 0.64 (P = 0.52)				
**Total (95% CI)**			**100.0%**	**0.78 [0.59, 1.02]**
Heterogeneity: Tau² = 0.09; Chi² = 22.08, df = 5 (P = 0.0005); I² = 77%				
Test for overall effect: Z = 1.81 (P = 0.07)				
Test for subgroup differences: Chi² = 0.44, df = 1 (P = 0.51), I² = 0%				

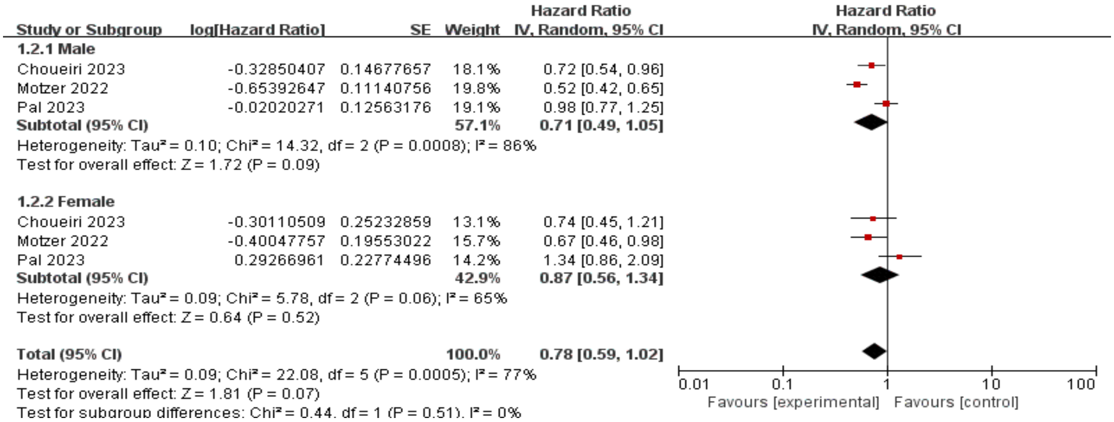

Study or Subgroup	log[Hazard Ratio]	SE	Weight	Hazard Ratio IV, Random, 95% CI
**1.3.1 Sarcomatoid features (Yes)**				
Choueiri 2023	-0.13926207	0.54291625	5.3%	0.87 [0.30, 2.52]
Motzer 2022	-1.07880966	0.29755992	14.6%	0.34 [0.19, 0.61]
**Subtotal (95% CI)**			**19.8%**	**0.49 [0.20, 1.19]**
Heterogeneity: Tau² = 0.25; Chi² = 2.30, df = 1 (P = 0.13); I² = 57%				
Test for overall effect: Z = 1.57 (P = 0.12)				
**1.3.2 Sarcomatoid features (No)**				
Choueiri 2023	-0.34249031	0.13123862	37.2%	0.71 [0.55, 0.92]
Motzer 2022	-0.51082562	0.10516449	43.0%	0.60 [0.49, 0.74]
**Subtotal (95% CI)**			**80.2%**	**0.64 [0.55, 0.75]**
Heterogeneity: Tau² = 0.00; Chi² = 1.00, df = 1 (P = 0.32); I² = 0%				
Test for overall effect: Z = 5.42 (P < 0.00001)				
**Total (95% CI)**			**100.0%**	**0.60 [0.46, 0.77]**
Heterogeneity: Tau² = 0.03; Chi² = 5.63, df = 3 (P = 0.13); I² = 47%				
Test for overall effect: Z = 3.91 (P < 0.0001)				
Test for subgroup differences: Chi² = 0.35, df = 1 (P = 0.56), I² = 0%				

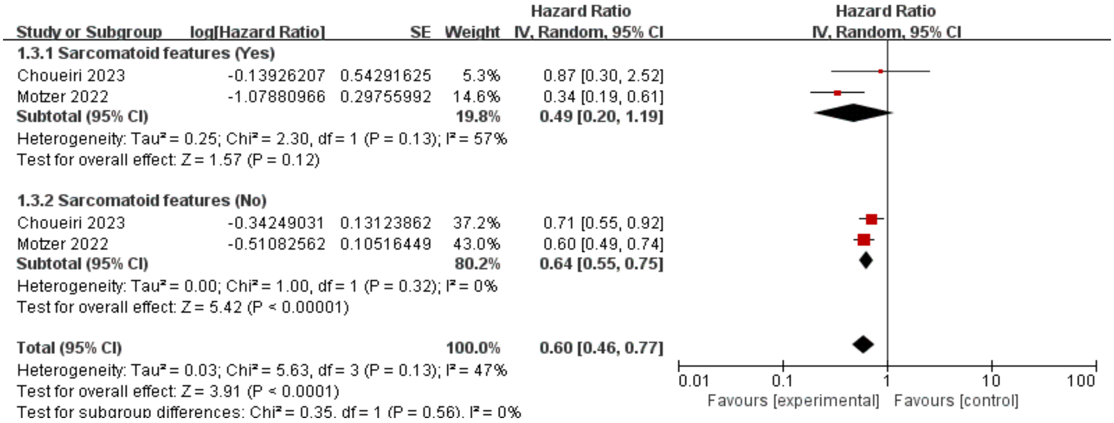

Study or Subgroup	log[Hazard Ratio]	SE	Weight	Hazard Ratio IV, Random, 95% CI
**1.4.1 Previous nephrectomy (Yes)**				
Choueiri 2023	-0.24846136	0.1582622	25.4%	0.78 [0.57, 1.06]
Motzer 2022	-0.65392647	0.1175549	36.2%	0.52 [0.41, 0.65]
**Subtotal (95% CI)**			**61.6%**	**0.63 [0.42, 0.93]**
Heterogeneity: Tau² = 0.06; Chi² = 4.23, df = 1 (P = 0.04); I² = 76%				
Test for overall effect: Z = 2.30 (P = 0.02)				
**1.4.2 Previous nephrectomy (No)**				
Choueiri 2023	-0.4780358	0.21166396	16.6%	0.62 [0.41, 0.94]
Motzer 2022	-0.41551544	0.17682326	21.8%	0.66 [0.47, 0.93]
**Subtotal (95% CI)**			**38.4%**	**0.64 [0.49, 0.84]**
Heterogeneity: Tau² = 0.00; Chi² = 0.05, df = 1 (P = 0.82); I² = 0%				
Test for overall effect: Z = 3.25 (P = 0.001)				
**Total (95% CI)**			**100.0%**	**0.63 [0.52, 0.76]**
Heterogeneity: Tau² = 0.01; Chi² = 4.45, df = 3 (P = 0.22); I² = 33%				
Test for overall effect: Z = 4.81 (P < 0.00001)				
Test for subgroup differences: Chi² = 0.01, df = 1 (P = 0.92), I² = 0%				

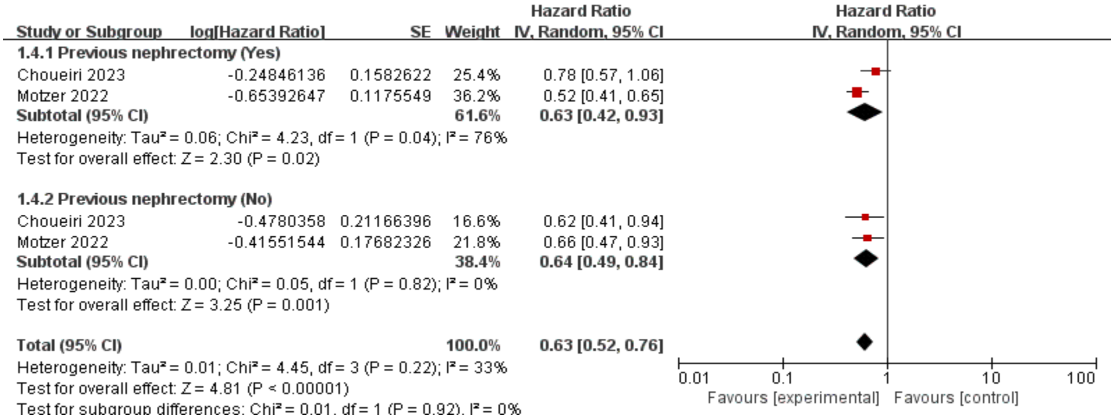

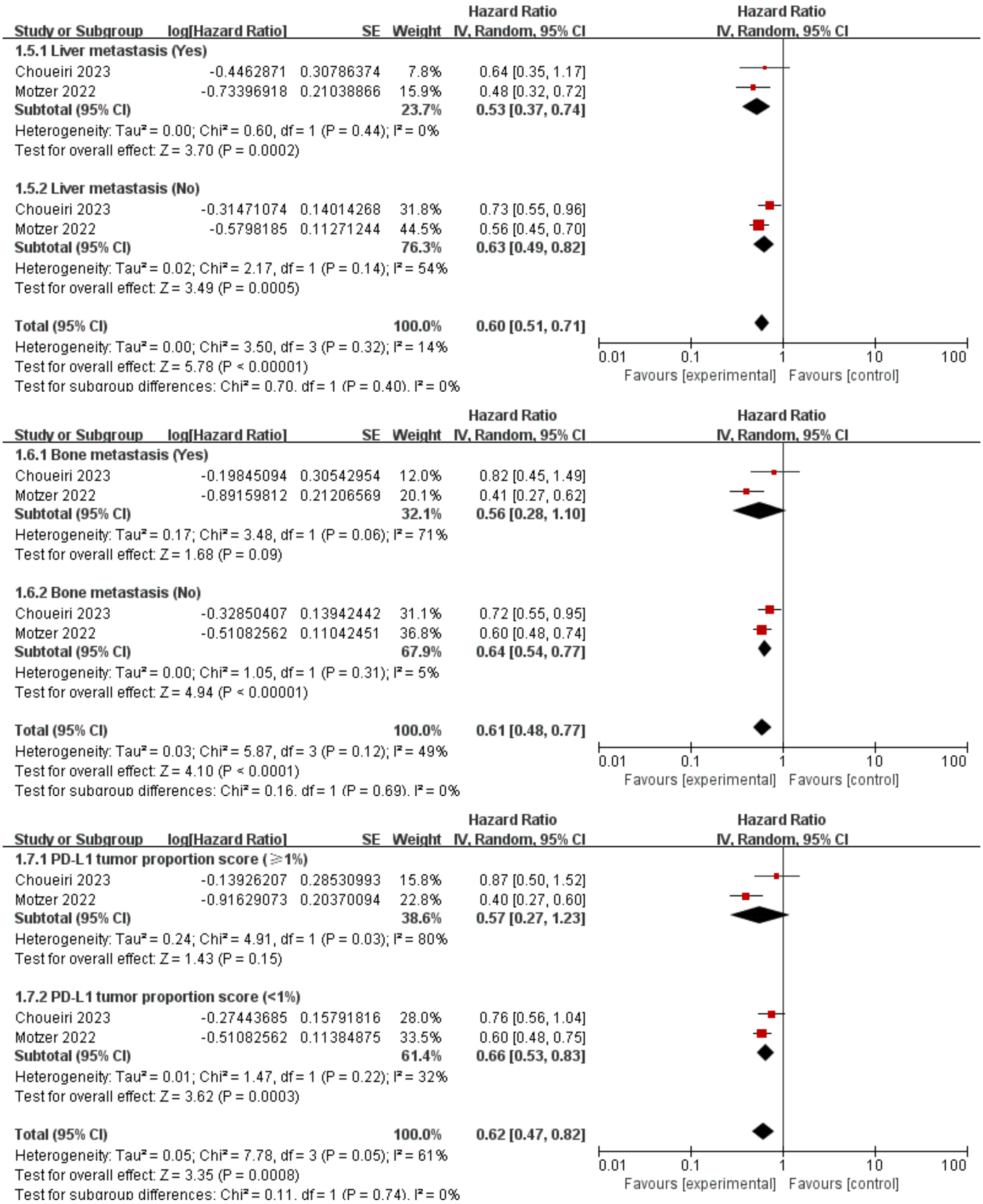

Supplementary Figure 1. Forest plot of the PFS subgroup analysis in randomized controlled trials.
